# Nitrogen Assimilation Related Genes in *Brassica* *napus*: Systematic Characterization and Expression Analysis Identified Hub Genes in Multiple Nutrient Stress Responses

**DOI:** 10.3390/plants10102160

**Published:** 2021-10-12

**Authors:** Xuyou He, Hao Zhang, Xiangsheng Ye, Juan Hong, Guangda Ding

**Affiliations:** 1 Key Laboratory of Arable Land Conservation (Middle and Lower Reaches of Yangtze River), Ministry of Agriculture and Rural Affairs/State Environmental Protection, Huazhong Agricultural University, Wuhan 430070, China; hxy0816@webmail.hzau.edu.cn (X.H.); hao.zhang@webmail.hzau.edu.cn (H.Z.); xiangshengye@mail.hzau.edu.cn (X.Y.); 2 Key Laboratory of Soil Health and Green Remediation, Ministry of Ecology and Environment/Micro-Element Research Center/College of Resources and Environment, Huazhong Agricultural University, Wuhan 430070, China; 3 Institute of Environment and Safety, Wuhan Academy of Agricultural Sciences, Wuhan 430070, China

**Keywords:** *Brassica napus*, nitrogen assimilation, genome-wide characterization, duplication profile, expression pattern, nutrient stress, protein structure

## Abstract

Nitrogen (N) is an essential macronutrient for plants. However, little is known about the molecular regulation of N assimilation in *Brassica napus*, one of the most important oil crops worldwide. Here, we carried out a comprehensive genome-wide analysis of the N assimilation related genes (NAGs) in *B. napus*. A total of 67 NAGs were identified encoding major enzymes involved in N assimilation, including asparagine synthetase (AS), glutamate dehydrogenase (GDH), glutamine oxoglutarate aminotransferase (GOGAT), glutamine synthetase (GS), nitrite reductase (NiR), nitrate reductase (NR). The syntenic analysis revealed that segmental duplication and whole-genome duplication were the main expansion pattern during gene evolution. Each NAG family showed different degrees of differentiation in characterization, gene structure, conserved motifs and cis-elements. Furthermore, diverse responses of NAG to multiple nutrient stresses were observed. Among them, more NAGs were regulated by N deficiency and ammonium toxicity than by phosphorus and potassium deprivations. Moreover, 12 hub genes responding to N starvation were identified, which may play vital roles in N utilization. Taken together, our results provide a basis for further functional research of NAGs in rapeseed N assimilation and also put forward new points in their responses to contrasting nutrient stresses.

## 1. Introduction

Nitrogen (N) supply affects metabolism, growth and yield of crops [1]. In the last 50 years, the amount of chemical N fertilizer applied to crops worldwide has risen dramatically [2]. However, only half of the applied N fertilizers can be taken up by crop plants, with much of the remainder lost to the environment [3]. Ecologically, the remaining N fertilizers in soils lead to devastating effects such as eutrophication [4]. Therefore, application of large amounts of N fertilizers is environmentally and economically unsustainable in the agriculture system. It is urgent to decrease the utilization of N fertilizers but without decreasing crop production, or to improve the N use efficiency (NUE) of plants.

NUE, which refers to the total biomass or grain yield per unit of N application, is composed of N uptake efficiency (NUpE) and N assimilation efficiency (NUtE) [5]. Except for environment factors such as soil conditions and temperature, the abilities of plant N uptake, assimilation and remobilization mediated by complex gene regulatory networks are the main constraints of NUE [5,6]. Ammonium (NH_4_^+^) and nitrate (NO_3_^−^) are the two mostly studied inorganic N forms, as they are often present in both natural and cropland soils at much higher levels than other inorganic N sources that crops can uptake and utilize [5]. The efficient uptake and transport of NH_4_^+^ and NO_3_^−^ mediated by ammonium and nitrate transporters contributes largely to NUpE of crops, and eventually promotes NUE [5]. Currently, many candidate genes have been identified and functionally characterized to be involved in regulating plant NUE. For instance, overexpression of nitrate transporter NRT1.1B significantly enhanced rice NUE [7].

In higher plants, N was absorbed in different forms, following by a series of assimilation processes. For NO_3_^−^, it was mainly delivered to shoot and reduced to NH_4_^+^ via nitrate reductase (NR) in the cytoplasm and nitrite reductase (NiR) in plastids and then utilized to synthesize amino acid (aa). However, for NH_4_^+^, most of them could be assimilated to aa directly through glutamine synthetase (GS)/glutamine-2-oxoglutarate aminotransferase (GOGAT) cycle in roots [5]. The prevailing forms of GS/GOGAT isoenzymes in leaves are chloroplastic GS2 and ferredoxin-GOGAT (Fd-GOGAT) located in plastids. In roots, the predominant GS/GOGAT isoenzymes, however, are GS1 and NADH-GOGAT located in the cytosol. GS is encoded by two classes of nuclear genes *GLN1* and *GLN2* which code for cytosolic GS1 and chloroplastic GS2, respectively [8]. Besides the GS/GOGAT cycle, other enzymes may also be involved in ammonium assimilation. Cytosolic asparagine synthetase (AS), which plays a crucial role in aa synthesis, could further catalyze glutamine and aspartate to form asparagine and glutamate [9]. Moreover, mitochondrial NADH-glutamate dehydrogenase (GDH) is usually involved in re-assimilation of excessive NH_4_^+^ released under intracellular hyper-ammonia condition by alternatively incorporating ammonium into glutamate [10].

The importance of the N assimilation related enzymes in regulating NUE has been highlighted by functional genomics and quantitative trait loci (QTL) approaches [5]. Excavating key genes involved in plant N assimilation can promote a better knowledge of the regulatory mechanisms, and is helpful to reduce the application of N fertilizer and to improve crop NUE. In *Arabidopsis*, the N assimilation related genes (NAGs) encoding AS, GDH, GOGAT, GS, NiR and NR has been fully characterized, including *ASN* [11], *GDH* [12], *GLT*/*GLU* [13], *GLN* [14], *NIR* [15] and *NIA* [16] families, respectively. QTL analysis of NUE in *Arabidopsis* suggested that at least 25 genes involved in N assimilation were located in the QTL regions, including genes encoding GS, GOGAT, NR and NiR [17]. Among them, genes coding for GS1 might be a key factor associated with NUE [18,19]. In addition, the mutation of *ASN2* resulted in NH_4_^+^ accumulation in plants [20]. The accumulation of aa, including alanine, g-aminobutyric acid and aspartic acid were badly affected in roots and leaves of *Arabidopsis gdh1/2/3* triple mutant [21]. Moreover, NAGs were not only closely related to N assimilation, but also participated in effectively improving plant adaptability to abiotic stresses. For example, overexpression of the GS genes significantly reduced H_2_O_2_ content and inhibited rice growth under cadmium stress environment [22]. The expression of *ASN2* was induced by the accumulation of ammonium in response to NaCl and cold stresses [23]. Likewise, the heterologous expression of a fungal *GDH* gene could increase NH_4_^+^ assimilation in rice, and thereby reduces its growth inhibition under NH_4_^+^ toxicity [24]. Interestingly, cytokinin could specifically enhance the activity of GDH3 in the roots of N-fed plants, which further indicates that some NAGs might have functional crosstalk on N metabolism and hormone signaling in different organs [25].

At present, NAGs have been systematically identified only in a few plant species such as apple [26] and *Populus* [27]. Except the model plants *Arabidopsis* and rice, our knowledge about the characteristics and function of NAGs in other crop plants is still limited. *Brassica napus* (genome AACC, 2n = 38), an important oil crop worldwide, is a recent allopolyploidy formed by the natural hybridization of two monogenomic diploids *B. oleracea* (Mediterranean cabbage, genome CC, 2n = 18) and *B. rapa* (Asian cabbage or turnip, genome AA, 2n = 20) [28]. A whole-genome triplication occurs shortly after polyploidization, which results in the genome size of *B. napus* being about two to six times larger than that of *B. rapa* and *B. oleracea* differentiated from *Arabidopsis*, as well as *Arabidopsis* itself [29]. Thereby, compared with *Arabidopsis*, NAGs may have obvious functional differentiation during the evolution of *B. napus*. Recent study showed that the NUE of *B. napus* varied among different genotypes [30]. However, the NUE of oilseed rape is relatively low compare with other crops, as *B. napus* accumulated approximately 6 kg N only to produce 0.1 t seeds [31]. Therefore, it is important to systematically characterize NAGs in the whole genome of rapeseed and to investigate their potential biological functions in regulating NUE. Here, we identified 67 NAGs in *B. napus*, as well as 30 and 34 in its two ancestors, *B. rapa* and *B. oleracea*, respectively. The duplication pattern and syntenic analysis between *Arabidopsis* and three *Brassica* species were performed to reveal the evolution process of NAGs. Moreover, the comprehensive genome-wide characterization of NAGs in oilseed rape were performed, including phylogenetic tree construction, gene structure and conserved motif analysis, chromosomal location analysis, potential cis-element identification and protein structure prediction. More importantly, we analyzed the response of NAGs to multiple nutrient stresses in detail based on the RNA-seq data. In a word, our results not only provide valuable information for further function dissection of NAGs in *B. napus*, but also reveal its potential in resisting other nutritional stresses except N.

## 2. Results

### 2.1. Genome-Wide Identification and Characterization of the NAGs in Brassica Species

To explore NAGs in *Brassica* species, the aa sequences collected from *Arabidopsis* NAGs encoding AS (*ASN1*-*ASN3*), GDH (*GDH1*-*GDH3*), GOGAT (*GLT1*, *GLU1* and *GLU2*), GS (*GLN1* and *GLN2*), NiR (*NIR1*) and NR (*NIA1* and *NIA2*) were used to perform BLASTP search against the genome databases of *B. rapa* (‘Chiifu-401′), *B. oleracea* (‘TO1000′) and *B. napus* (‘Darmor-*bzh*’). In general, we retrieved 30, 34 and 67 NAGs in *B. rapa*, *B. oleracea* and *B. napus*, respectively (Table 1). Among them, the *GLN1* subfamily encoding glutamine synthetase has the most members, followed by *GDH2* and *NIA1*. On the contrary, the *NIA2* subfamily has only one member in *Arabidopsis*, while it was not observed in *Brassica* species. Therefore, large changes of evolution and selection occurred in each NAG subfamily during *Brassica* evolution. In addition, the number of most NAG subfamilies in *B. napus* were similar to the sum of the two other *Brassica* species, owing to the heterotetraploization of *B. napus* from *B. rapa* and *B. oleracea* [28]. These results indicate that each NAG subfamily might have different expansion modes to adapt to the changes of external N concentration and promote N assimilation during the evolution of *Brassica* species.

**Figure 1:**
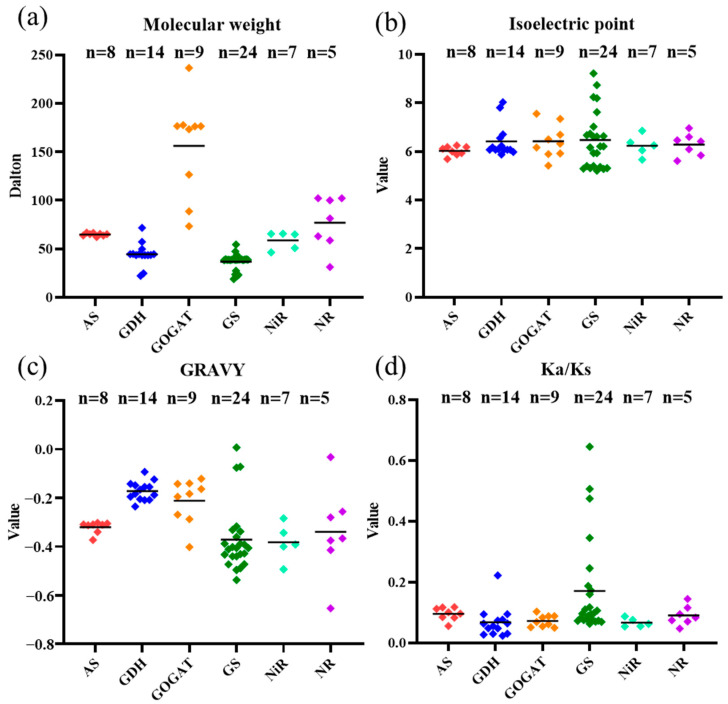
Molecular characterization of the NAGs in *Brassica napus*. (**a**) Molecular weight; (**b**) theoretical isoelectric point; (**c**) grand average of hydropathy; (**d**) nonsynonymous (Ka)/synonymous (Ks) values. Genes from the same group are shown by the same color. AS, asparagine synthetase; GDH, glutamate dehydrogenase; GOGAT, glutamine oxoglutarate aminotransferase; GS, glutamine synthetase; NiR, nitrite reductase; NR, nitrate reductase.

Then, the physicochemical properties of the NAGs in *B. napus* were further analyzed (Figure 1, Table S1). The aa length was conserved in proteins for AS, GDH and NiR, which ranged from 555 to 592, 203 to 652 and 411 to 585, respectively (Table S1). Although the aa length for GDH varied largely, most of its members (78.6%) had 403~457 aa. On the contrary, the aa length of proteins for GOGAT, GS and NR were 665~2162, 169~489 and 279~911, respectively (Table S1). Likewise, the gene length varied from 510 bp to 7010 bp, with a large variation in genes encoding GOGAT, GS and NR. Accordingly, the molecular weight (MW), isoelectric point (pI) and grand average of hydropathy (GRAVY) values of the NAGs in *B. napus* revealed various degrees of differentiation (Figure 1, Table S1). The results showed that, compared with other gene families, the MW of the GOGAT family had more divergence (73.45~236.59 kDa), and seven GOGAT family members were more than 100 kDa (Figure 1a). For pI, 85.1% of the NAGs were less than 7, indicating that they were acidic proteins (Figure 1b, Table S1). Furthermore, the predicted GRAVY values based on protein sequences suggested that they were hydrophilic in nature with GRAVY < 0, except BnaC05.GLN1;3a (Figure 1c, Table S1). Similar to the aa length, the GRAVY values of genes encoding GS and NR changed greatly as well (Figure 1c). These results further supported the intense divergence of NAGs in rapeseed during evolution. Furthermore, subcellular location prediction indicated that most of the NAGs located in cytoplasm and chloroplast, while a few genes were also found to locate in cytoskeleton, golgi apparatus, mitochondria and nucleus (Table S1).

### 2.2. Phylogenetic Analysis of the NARGs in Brassica napus

**Figure 2:**
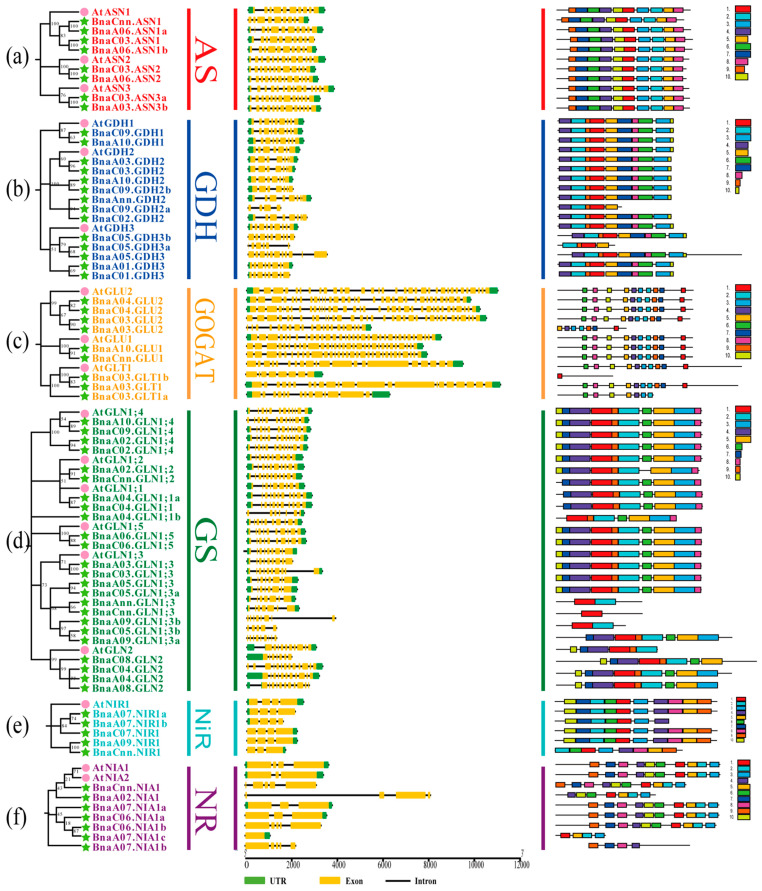
Phylogenetic relationships, gene structure and architecture of conserved protein motifs in the nitrogen assimilation-related genes of *Brassica napus* and *Arabidopsis thaliana*. The analysis includes AS (**a**), GDH (**b**), GOGAT (**c**), GS (**d**), NiR (**e**) and NR (**f**) families. The MEGA7.0 software is used to construct the phylogenetic trees with the NJ method. The intron analysis is carried out with genomic and coding sequences of the *Arabidopsis* and rapeseed NAGs. Exon-intron structure is indicated by yellow rectangles and black lines, respectively. The untranslated region (UTR) is shown by green box. Conserved motifs are detected using MEME, and the boxes with different colors indicate different conserved motifs. The motif sequence information is provided in Table S2. AS, Asparagine synthetase; GDH, Glutamate dehydrogenase; GOGAT, glutamine oxoglutarate aminotransferase; GS, glutamine synthetase; NiR, nitrite reductase; NR, nitrate reductase.

A total of 14 and 67 NAGs in *Arabidopsis* and *B. napus* were used to construct a comprehensive phylogenetic tree using neighbor-joining (NJ) method (Figure 2). To better classify these genes and investigate their evolutionary relationships, we divided them into AS, GDH, GOGAT, GS, NiR and NR families and renamed all the rapeseed NAG homologues according to the homology with *Arabidopsis* NAGs following the international nomenclature for *B. napus* genes (Figure 2, Table S1). In detail, AS family contained 11 genes (3 *AtASNs* and 8 *BnaASNs*), GDH family contained 17 genes (3 *AtGDHs* and 14 *BnaGDHs*), GOGAT family contained 12 genes (1 *AtGLT1*, 2 *AtGLUs*, 3 *BnaGLT1s* and 6 *BnaGLUs*), NiR family contained 6 genes (1 *AtNIR1* and 5 *BnaNIR1s*) and NR family included 9 genes (2 *AtNIAs* and 7 *BnaNIA1s*). The remaining 30 genes belonged to the GS family (6 *AtGLNs* and 24 *BnaGLNs*) (Figure 2). Within each NAG family, each *Arabidopsis* gene was closely related to more than two genes in *B. napus*. Among them, *AtGLN1;3* had nine homologues in oilseed rape, owing to the gene duplication events in the GS family during evolution. However, we did not find any genes homologous to *AtNIA2* in *B. napus*. In general, genome-wide and intrafamilial expansion may be the main reasons for these phenomena.

### 2.3. Gene Structure and Conserved Motif Analysis of the NAGs in Brassica napus

Deletion or insertion of exon-intron structure might lead to divergence in coding regions and finally result in functional diversification. In another words, exon-intron structure is the important feature during evolutionary process [32]. Therefore, the gene structure of the NAGs in *Arabidopsis* and *B. napus* was further investigated (Figure 2, Table S1). The results showed that the intron number of the GOGAT family ranged from 7 to 35 with the average of 25, while the NIR and NR families were only 0 to 4 with the average of 3 (Table S1). The intron number of the AS, GDH and GS family genes ranged from 6 to 12. It is obvious that the NAGs within the same family had similar gene structure with their orthologues in *Arabidopsis* (Figure 2). However, a few genes displayed strong differentiation in gene structure, especially the *GLT1*, *GLN1;3* and *NIA1* subgroups. The gene size and intron-exon structure of these genes in *B. napus* were significantly different from those of the *Arabidopsis* homologous genes, suggesting that they might have additional or novel physiological functions.

Furthermore, the conservative motifs of the NAGs in *Arabidopsis* and *B. napus* were obtained based on their aa sequences via MEME software (Figure 2, Table S2). A total of 10 motifs were identified in each family, ranging from 4 to 50 aa. Similar to gene structure, most genes with close phylogenetic relationships had high similarity in conserved motif composition. In particular, all genes in the AS family contained motif 1~10, indicating that this family might have highly conserved functions or even functional redundancy among genes. It is worth noting that the conserved motif organizations of *GLT1*, *GLN1;3* and *NIA1* families also showed significant divergence. In *GLT1* family, only Motif 1 was identified in *BnaC03.NLT1b*, while Motif 1, Motif 7 and Motif 9 were not found in *BnaC03.GLT1a*. Four *BnaGLN1;3s* had Motif 4, Motif 7 and Motif 10, and others only contained Motif 1 or Motif 2. Additionally, the motifs of *BnaA07.NIAb* and *BnaA07.NIAc* were observed at N-terminal and C-terminal in *AtNIA1*, respectively. Above all, these results further support our hypothesis of functional differentiation for the NAGs in oilseed rape.

### 2.4. Chromosomal Distribution and Syntenic Analysis of the NAGs in Brassica napus

The 67 NAGs in *B. napus* displayed an uneven distribution on 19 chromosomes. Among them, 34 genes located on the A subgenome and 33 genes located on the C subgenome, respectively (Figure S1). Chromosome C03 contained the most member with eight NAGs, while chromosomes A01 and C01 had the fewest number with only one NAG. In detail, many genes coding for AS and GOGAT located on chromosome C03, while more NIR and NR family members located on chromosome A07 than other chromosomes. Many GDH and GS family members located on chromosomes C09 and A04, respectively. Additionally, the location of *BnaA05.GDH3* and *BnaA08.GLN2* on chromosomes A05 and A08 were not identified, and eight rapeseed NAGs could not be mapped to a specific chromosome.

**Figure 3:**
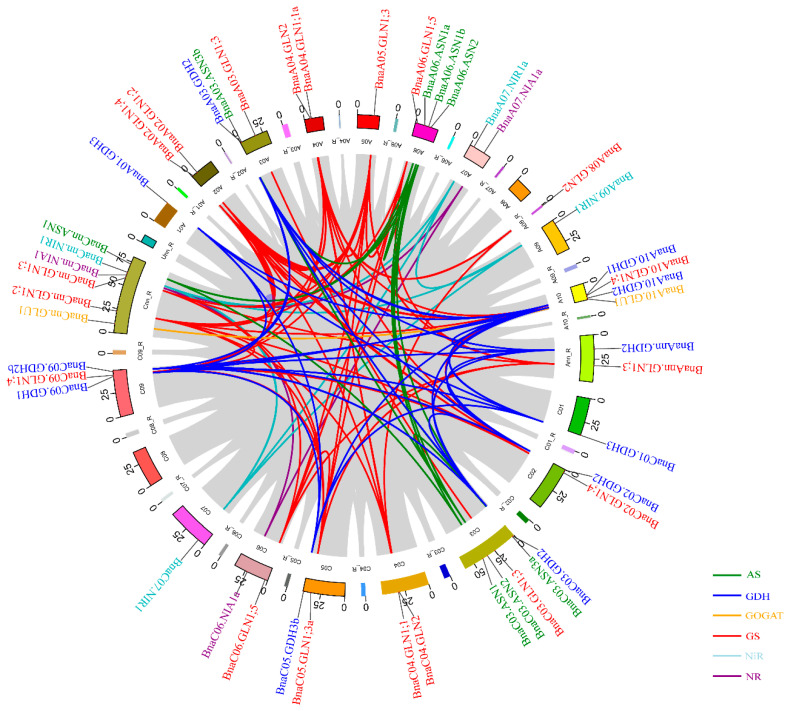
Schematic representations for the chromosomal distribution and interchromosomal relationships of the nitrogen assimilation related genes in *Brassica napus*. Gray lines indicate all syntenic blocks in the *B. napus* genome, and the colored lines indicate syntenic gene pairs of different gene families. The chromosome number is shown at the bottom of each chromosome. R, random. The number besides the chromosome indicates the length (Mb) of it.

A tandem duplication event was defined as adjacent genes on chromosomal region within 200 kb [33]. We found that three gene pairs (*BnaA07.NIAb* and *BnaA07.NIAc*, *BnaC03.GLT1a* and *BnaC03.GLT1b*, *BnaC05.GDH3a* and *BnaC05.GDHb*) within the same family were extremely adjacent to each other on the corresponding chromosomes (Figure S1). Therefore, we analyzed the gene duplication patterns of each family based on their coding sequences using MCScanX, including tandem and segmental duplications. A total of 12, 25, 50, 6 and 3 segmental duplication events were identified for the gene families encoding AS, GDH, GS, NiR and NR, respectively. However, only one gene pair was observed for the GOGAT family (Figure 3).

**Figure 4:**
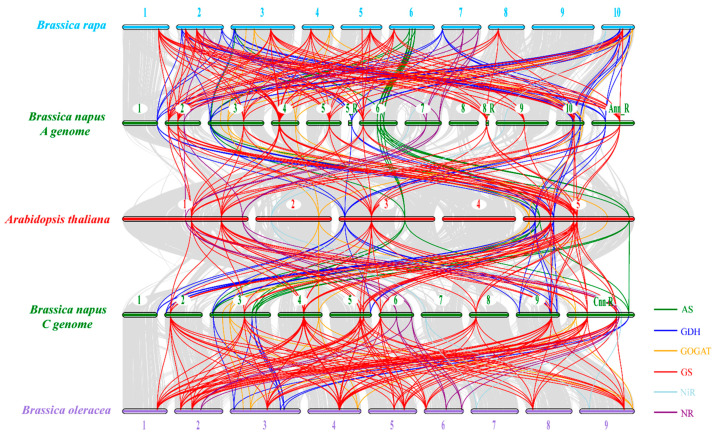
Synteny analysis of the nitrogen assimilation-related genes in *Brassica napus*, *B. rapa*, *B. oleracea* and *Arabidopsis thaliana* chromosomes. Gray lines in the background indicate all syntenic blocks between the genomes of *B. napus* and other species, while the lines with different colors indicate syntenic gene pairs of each family. The green, blue, yellow, red, cyan and purple color lines indicate asparagine synthetase (AS), glutamate dehydrogenase (GDH), glutamine oxoglutarate aminotransferase (GOGAT), glutamine synthetase (GS), nitrite reductase (NiR), nitrate reductase (NR) family gene pairs, respectively.

Furthermore, we constructed a syntenic map of *Arabidopsis* and three *Brassica* species (*B. rapa*, *B. oleracea* and *B. napus*) to further uncover the evolution processes of the NAGs. The results revealed that there were strong orthologs of the NAGs among *B. napus*, *B. rapa*, *B. oleracea* and *Arabidopsis*, especially in genes coding for the GS family (Figure 4). In the A subgenome of *B. napus*, there were 105 and 356 pairs of collinear relationships with *Arabidopsis* and *B. rapa*, respectively. Among them, 58 (55.2%) and 199 (55.9%) were observed in the genes encoding GS. Moreover, there were 112 and 345 pairs of syntenic relationships in the C subgenome of *B. napus* with *Arabidopsis* and *B. oleracea*, respectively. Among them, 60 (53.6%) and 120 (60.0%) were found in the GS gene family. Interestingly, the syntenic relationships of the AS, GOGAT and NR family genes in *B. napus* with other species were mainly concentrated on one or two chromosomes of the two subgenomes (Figure 4). Taken together, the results of the syntenic analysis indicated that segmental duplication and whole-genome duplication might be the main driving force for the expansion of the NAGs in oilseed rape.

Moreover, we estimated the nonsynonymous (Ka), synonymous (Ks) and Ka/Ks ratios of the NAGs between *B. napus* and *Arabidopsis*, which were used as diagnostic criterion of sequence evolution (Figure 1d, Table S3). A mutation that altered a protein is much less likely to be different between two species than silent ones, which means that most of the choices eliminate harmful mutations and keep the protein as it is [34]. Likewise, our results showed that all the Ka/Ks ratios of the NAGs were less than 1, suggesting that the evolutionary processes of the NAGs between *B. napus* and *Arabidopsis* were mainly affected by purifying selective pressure.

### 2.5. Cis-Elements Analysis in the Promoter Regions of the NAGs in Brassica napus

**Figure 5:**
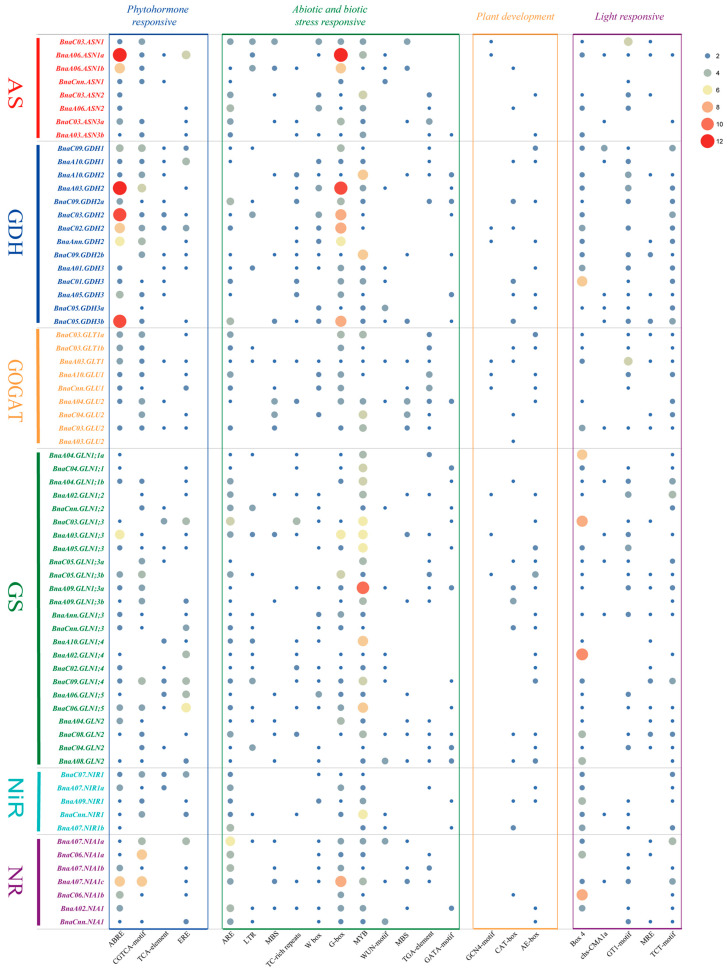
The cis-acting regulatory elements in the promoter regions of the rapeseed nitrogen assimilation-related genes. The blue, green, orange and purple blocks represent phytohormone, abiotic and biotic stress, plant development, and light response cis-elements, respectively. The number of cis-elements is indicated by different colors and circle size.

In order to better understand the potential function and transcriptional regulation of the rapeseed NAGs, the 2.0-kb upstream promoter sequences were used to detect cis-elements in PlantCARE (Figure 5). In addition to the necessary components including TATA-Box and CAAT-Box, a large number of cis-elements were identified in the promoter regions of the rapeseed NAGs. The identified cis-elements can be divided into four categories: (1) The phytohormone response related cis-elements, mainly including ABRE, CGTCA-motif, TCA-element and ERE. Among them, ABRE element, which is mainly involved in the regulation of abscisic acid (ABA) signaling pathway [35], was the mostly identified cis-element in the promoters of the rapeseed NAGs, especially in the AS and GDH families; (2) The abiotic and biotic stress response related cis-elements, which were the mostly observed in the rapeseed NAG promoters. For instance, LTR and MBS elements play important roles in response to low-temperature and drought stress, while MYB element and GATA-motif mainly regulate plants tolerance to low N stress [36,37]; (3) Cis-elements involved in plant development; (4) Light response related cis-elements. In a word, these results indicate that the *B. napus* NAGs might have significant biological functions in abiotic and biotic stress response.

### 2.6. Expression Profiles of the Rapeseed NAGs in Response to Multiple Nutrient Stresses

**Figure 6:**
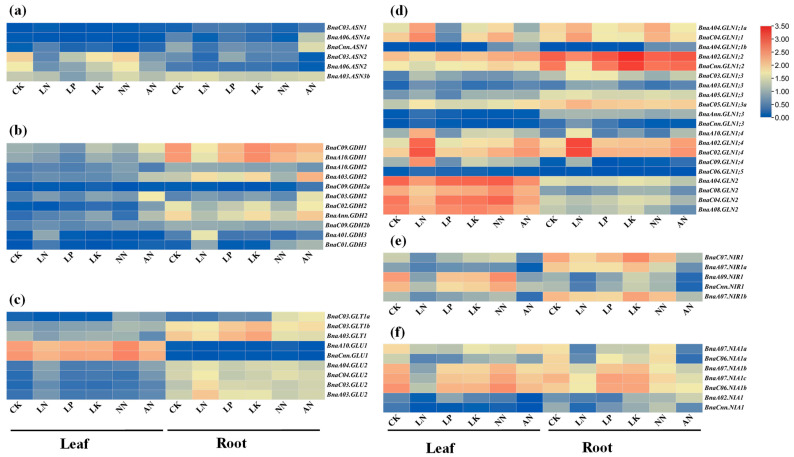
Expression profiles of the genes involved in nitrogen (N) assimilation in leaf and root of *Brassica napus* under N, phosphorus (P), potassium (K) deficiencies and ammonium (NH_4_^+^) toxicity. (**a**) The AS family genes; (**b**) The GDH family genes; (**c**) The GOGAT family genes; (**d**) The GS family genes; (**e**) The NiR family genes; (**f**) The NR family genes. For nutrient starvation treatments, 14-day-old seedlings were exposed to nutrient solution free of N, P and K for seven days. For the NH_4_^+^ toxicity treatments, 14-day-old seedlings were treated with nutrient solution containing 6 mM NH_4_Cl or 6 mM NaNO_3_ (control) for nine days. The fully expanded leaf and root were sampled separately for RNA-seq analysis. CK, sufficient nutrient supply. LN, N deficiency. LP, P deficiency. LK, K deficiency. NN, NO_3_^−^-N. AN, NH_4_^+^-N. The colored scale was shown on the right side. Heat maps of gene expression profiles were generated using TBtools after data normalization (Z-score).

Cis-element analysis revealed that most rapeseed NAGs harbored abundant abiotic stress-related elements in the promoter regions (Figure 5). Therefore, we further explored the expression patterns of the *B. napus* NAGs in response to different nutritional stresses including N, phosphorus (P), potassium (K) deficiencies and ammonium (NH_4_^+^) toxicity based on the RNA-seq data (Figure 6, Table S4). Generally, the results revealed that the differentially expressed genes (DEGs) involved in N assimilation in *B. napus* had tissue-specific expression patterns with different responses to nutrient deficiencies. Under normal nutrient supply, approximately 36 (62.1%) DEGs were mainly expressed in root, while only 16 (27.6%) DEGs were preferentially expressed in leaf (Figure 6). In particular, all genes encoding GDH were principally expressed in root (Figure 6b). Among all the DEGs, the expression levels of 25, 11, 17 and 19 genes in leaf were induced by N, P, K limitations and NH_4_^+^ toxicity, respectively, while 25, 23, 13 and 26 genes were down-regulated in the same conditions. In root, 15, seven, 21 and 21 DEGs were significantly up-regulated by N, P, K deprivations and NH_4_^+^ toxicity, respectively, and 18, 13, eight and 22 DEGs were inhibited in the same conditions. It is obvious that the responses of the GS and NR genes to different stresses were more disordered due to the significant differentiation of gene structure and conserved domains compared with other gene families (Figure 6d,f). However, the expression pattern is relatively conservative in each subfamily, although the overall expression of the GS family genes was highly differentiated (Figure 6d). Similar phenomenon was observed in the expression pattern of the GOGAT family (Figure 6c).

**Figure 7:**
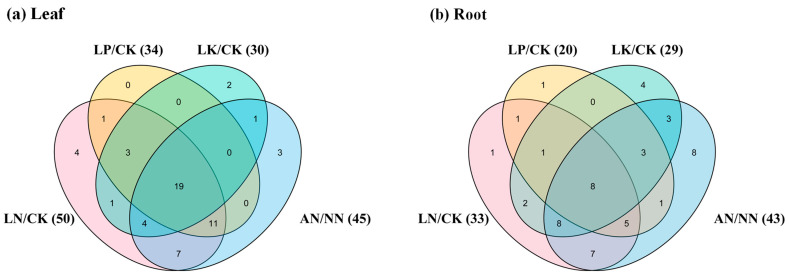
Venn diagram showing the transcriptional responses of the nitrogen assimilation-related genes in the leaf (**a**) and root (**b**) of *Brassica napus* under diverse nutrient deficiencies. Each color represents different treatment, and the number in brackets represents the differentially expressed genes between control and the corresponding treatment. CK, control; LN, nitrogen free condition; LP, phosphorus free condition; LK, potassium free condition; NN, nitrate-fed only; AN, ammonium-fed only.

In addition, we found that the NAGs in *B. napus* could respond to multiple nutrient stresses simultaneously (Figure 7). Only four, two and three genes had specific responses to N and K starvations and ammonium toxicity in leaf, respectively. No genes changed under P stress condition in leaf (Figure 7a). In root, one, one, four and eight genes responded to N, P, and K starvations and ammonium toxicity, respectively (Figure 7b). Furthermore, the expression levels of 19 and eight NAGs were regulated simultaneously across all the environments in leaf and root, respectively (Figure 7). These results further convinced the functional diversity of the NAGs in *B. napus*. Meanwhile, 77.8% (7/9) genes encoding GOGAT responded to all the conditions in leaf or root. Interestingly, four genes (*BnaC03.ASN2*, *BnaA10.GLU1*, *BnaC03.GLU2* and *BnaCnn.NIR1*) were affected dramatically by diverse nutrient stresses simultaneously in different tissues.

### 2.7. Coexpression Relationship Analysis and Gene Expression Verification of the NAGs in Brassica napus

**Figure 8:**
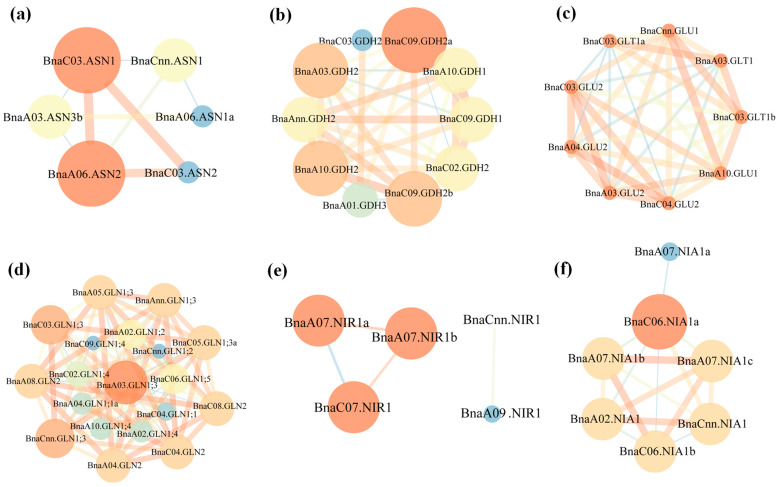
Coexpression networks of the genes involved in nitrogen assimilation in Brassica napus. (**a**) The AS family genes; (**b**) The GDH family genes; (**c**) The GOGAT family genes; (**d**) The GS family genes; (**e**) The NiR family genes; (**f**) The NR family genes. Each cycle node represents a gene, and the size of the node represents the power of the interrelation among the nodes by degree value. The width of the lines indicates the strength of the interactions be-tween two genes.

To further understand the molecular mechanisms of the rapeseed NAGs in response to N stress, the gene interaction networks were constructed based on the fragments per kilobase million (FPKM) values of the RNA-seq data under N limitation condition. A total of nine, 34, 36, 93, 4 and 16 coexpression relationships were identified for the genes coding for AS, GDH, GOGAT, GS, NiR and NR, respectively (Figure 8). Except the GOGAT family genes, there were significant differences in the gene interaction intensity of other families. Consistent with the results of gene structure and motif analysis, the interactions were greatly sophisticated with each other, and *BnaGLN1;3* subfamily appeared to be in the core position within the GS family (Figure 8d). Interestingly, although we found that all the AS family genes were conservative in the coding region, their coexpression relationships emerged the opposite situation. This may be due to the large variations in the promoter regions of the AS family genes. For the GDH, NiR and NR families, most of the genes had strong interaction relationship intensity (Figure 8b–f).

**Figure 9:**
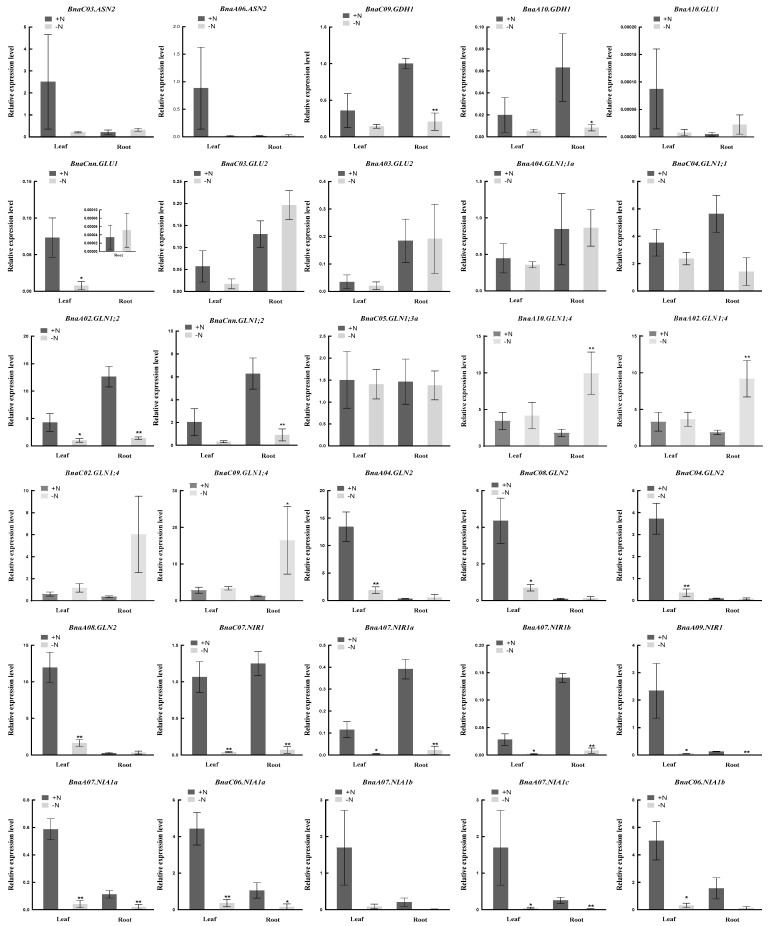
The expression profiles of 30 genes involved in nitrogen assimilation in the leaf and root of *Brassica napus* under nitrogen (N) stress by qRT-PCR. Seedlings of 14 days old were exposed to N-free nutrient solution for six days. The root and leaf were sampled separately for RNA extraction. +N, sufficient nutrient supply (6 mM N); -N, N stress (0 mM N) condition. * and ** indicate significant difference at *p* < 0.05 and *p* < 0.01 by Student’s *t*-test, respectively.

In addition, we selected 30 rapeseed NAGs according to the gene expression abundance and interaction networks to further verify their expression patterns under N deficient condition using quantitative real-time PCR (qRT-PCR) (Figure 9). The results demonstrated convincingly that the genes in each subfamily had the same response style to N stress, which was consistent with the changes in the RNA-seq data. Nearly all the NAGs were down-regulated or unchanged under N deprivation, while only four *BnaGLN1;4s* were significantly induced in root. Moreover, two *BnaASN2s*, two *BnaGLU1s*, four *BnaGLN2s*, one *BnaNIR1* and five *BnaNIA1s* were expressed constructively in leaf, while the others were expressed mainly in root (except *BnaA04.GLN1;1a*, *BnaC04.GLN1;1*, *BnaC05.GLN1;3a* and *BnaC07.NIR1*). Thus, these evidences further convinced the expression specificity of the NAGs in *B. napus*. According to the fold changes between normal and N-free conditions, we chose 12 hub genes associated with N assimilation and extracted the information from cis-element analysis (Figure S2). We observed that the N stress response and hormone response related cis-elements were distributed abundantly in the promoter regions of the *B. napus* NAGs, which further confirmed that these genes might play important roles in regulating NUE or hormone interaction in *B. napus*.

### 2.8. D Protein Structure Analysis of the Hub NAGs in Brassica napus

**Figure 10:**
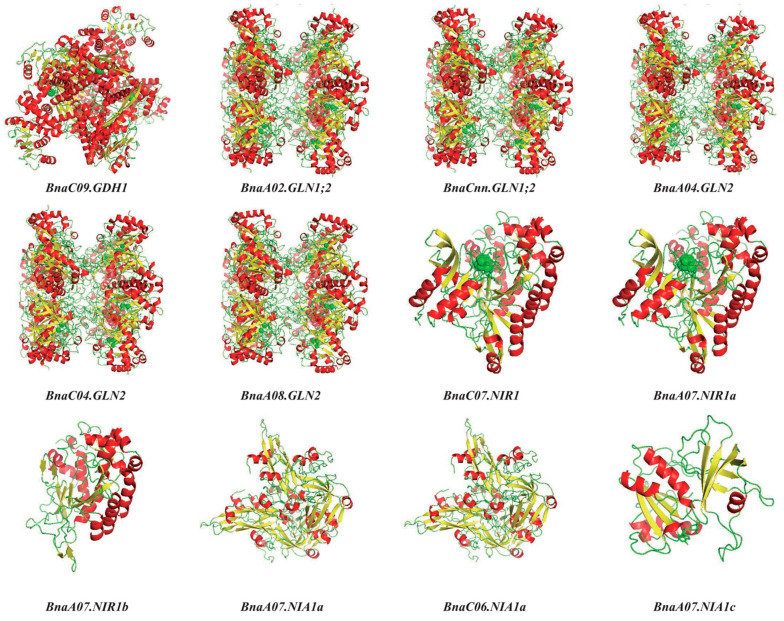
Predicted 3D structure of 12 core proteins associated with N assimilation using the SWISS-MODEL online software. The structure image is generated using the pymol software. The red, yellow and green color indicates alpha helix, beta turn and random coil, respectively.

The 3D structure of 12 core proteins associated with N assimilation were predicted based on homology modeling using SWISS-MODEL online software. Ramachandran plots of all 3D protein models showed that more than 90% of the residues were scattered in the red core region (the ideal conformation space), indicating that the models were highly reliable (Figure S3). Combined with the prediction of the secondary structure, the alpha helix, extended strand, beta turn and random coil of all the proteins ranged from 24.01–48.66%, 16.04–25.45%, 4.44–7.17% and 28.22–49.96%, respectively (Figure 10, Table S5). Therefore, each protein structure was dominated by alpha helix, while the proportion of beta turn was the smallest. Additionally, the 3D protein structures of *BnaA04.GLN2* and *BnaC04.GLN2* were identical, indicating that they might be redundant in molecular function. It is noteworthy that there were aggregates composed of a large number random coil in *BnaC07.NIR1* and *BnaA07.NIRa*, which could play an important role as special structures. Above all, the 3D structure of these proteins laid a foundation for exploring their biological functions.

## 3. Discussion

Ameliorating N assimilation efficiency is of great importance in improving NUE in plant [38]. The activities of several N assimilation-related enzymes such as GS and NR could directly reflect N level in plants [39]. Therefore, we comprehensively identified six NAG families in *Brassica* species. A total of 30, 34 and 67 NAGs were detected in *B. rapa*, *B. oleracea* and *B. napus*, respectively, and unevenly distributed across the chromosomes (Figure S1, Table 1). Consistent with the evolution of *Brassica* species, the number of NAGs in *B. napus* was similar to the sum of the NAGs both in *B. rapa* and *B. oleracea* (Table 1). The expansion pattern differed among different gene family, and *NIA2* subfamily disappeared during the evolution of *Brassica* species (Table 1). In *Arabidopsis*, *NIA1* and *NIA2* isoforms were not functionally equivalent with the former [40]. The NR family among *B. napus* and *Arabidopsis* were mainly affected by purifying selective pressure, which suggested that *NIA2* may be abandoned in order to optimize the genome during the *Brassica* species evolution (Figure 1d).

Tandem duplication, segmental duplication and whole-genome duplication were considered as three main driving forces for gene expansion [32]. In the present study, the gene expansion pattern of the AS, GDH, GS, NiR and NR family was mainly segmental duplication and whole-genome duplication, while only GOGAT family expansion seemed to be driven mainly by the latter (Figure 3; Figure 4). One of the most important effects of segmental duplication and whole-genome duplication was to promote complex rearrangement in the gene evolution process, including deletion, duplication, conversion, inversion, and translocation [41]. Here, we found that some NAGs in *B. napus* showed significant divergence compared with *Arabidopsis*, especially the GOGAT and NR family members (Figure 2). Although the GS family had the largest number of members, most of them were involved in segmental duplication and whole-genome duplication events, and were relatively conservative except the *GLN1;3* subfamily (Figure 2). In addition, duplication genes played potential roles in clarifying the genetic relationships that were difficult to be revealed by standard single gene mutation analysis [42]. For example, the gene structure and motif composition of the AS family members were intensely conservative, which suggested that they might be redundant in function (Figure 2).

Cis-elements play important roles in regulating gene expression in different biological processes. For example, *ZmNLP5* could directly bind to the cis-element on *ZmNIR1.1* promoter to modulate N response in maize [43]. In rice, *OsMYB55* binds to the *OsGS1;2* promoter and mediates aa metabolism at high temperature [44]. Here, we identified a large number of abiotic and biotic stress response related cis-elements in the promoter regions of the rapeseed NAGs (Figure 5), especially G-box and MYB cis-elements, which are involved in N response in plants [36,45]. In addition, it is reported that several NAGs are regulated by hormone and light [27,46]. Similarly, the phytohormone and light related cis-elements such as ABRE, ERE, GT1-motif and TCT-motif were unevenly distributed in the promoter regions of the rapeseed NAGs (Figure 5). In general, these results suggested that NAGs might be conservative in function between *B. napus* and *Arabidopsis*. However, the cis-element G-box only appeared in the promoters of *BnaA06.ASN1a* and *BnaA06.ASN1b*, indicating that functional differentiation also existed within each rapeseed NAG family (Figure 5).

To date, the function of NAGs in improving plant NUE had been fully explored. *Arabidopsis glt1* mutant had specific defects in non-photorespiratory ammonium assimilation and glutamate synthesis, while *NIR1* was an important target in controlling NO homeostasis [13,15]. In plant with single NiR such as *Arabidopsis* and *B. napus*, the nitrite reduction to ammonium is likely a key step to determine plant growth rate [15]. Our RNA-seq data analysis revealed that 82.6% (57/69) NAGs responded significantly to N deficiency and NH_4_^+^ toxicity (Figure 6, Table S4), indicating their vital roles in rapeseed N assimilation and utilization. In addition, NAGs in plants might be regulated by different abiotic stresses. Previous reports showed that the GDH activity showed a positive synergistic effect mediated by normal K supply, but it appeared an uncompetitive inhibition under high K condition [47]. Here, we explored the regulation of P and K deprivations on NAG expression. We found that the expression of 58.0% (40/69) and 60.9% (42/69) NAGs in different tissues changed significantly under P and K deficiencies, respectively. These results clearly indicated that the rapeseed NAGs might be involved in P and K homeostasis in *B. napus* (Figure 6, Table S4). Under low N condition, the NR activities and other N assimilation related enzymes were significantly inhibited in *B. napus* [48]. Here, we observed that the expression abundance of most *NAGs*, including 12 hub genes, decreased significantly under N deficiency (Figure 6; Figure 9). Moreover, NH_4_^+^ toxicity could inhibit the GS and GOGAT activities in *B. napus* roots [49], and most of the coding genes were down-regulated by NH_4_^+^ toxicity (Figure 6). All genes encoding NiR and NR were suppressed significantly by NH_4_^+^ toxicity as well, which might decrease nitrate reduction to produce less NH_4_^+^ (Figure 6e,f). In a word, *B. napus* might inhibit N assimilation related pathways in response to low N and NH_4_^+^ toxicity. However, the lack of P and K also changed the expression of some genes encoding NIR, NR and GDH, which may indirectly affect the assimilation process via the inhibited N uptake [50,51].

The genomic gene interaction network based on large-scale gene expression data provided an effective method to quickly uncover the gene interaction and molecular mechanism of various biological processes and developmental programs [52]. Obviously, there were significant divergence in the interaction coefficients among most NAGs in *B. napus* (Figure 8), indicating their specific roles in N assimilation. Thus, we identified 12 hub NAGs based on the expression profiles and coexpression network analysis to further reveal their potential biological functions (Figure 8; Figure 9). Likewise, except the N stress response cis-element, some phytohormone response elements were also observed in the promoter regions of 12 core rapeseed NAGs, indicative of the complex regulatory networks between N assimilation and phytohormones (Figure S2). A model with high quality was required to have more than 90% of the residues in the preferred area [53]. The 3D structure model fully illustrated the protein structures of the 12 core NAGs, which provided important clues for further exploring the biological functions of the NAG family in *B. napus* (Figure 10 and Figure S3).

## 4. Materials and Methods

### 4.1. Identification of NAGs in Brassica napus

Firstly, the aa sequences of NAGs were retrieved and obtained from the *Arabidopsis* Information Resource (TAIR) database (https://www.Arabidopsis.org/, accessed on 28 August 2019), and then were used for a BLASTP search in the *B. napus* genome database (http://www.genoscope.cns.fr/brassicanapus/, accessed on 28 August 2019) [54]. Redundant sequences were removed manually. The Pfam database (http://pfam.xfam.org/, accessed on 28 August 2019) was used to identify standard genes with E-value ≤ 0.001. To confirm the accuracy of the genes, all candidate genes were aligned with *Arabidopsis* homologous genes. The genomic DNA, cDNA, CDS and protein sequences of the NAGs were obtained from the *B. napus* genome database [28]. The NAGs in *B. rapa* and *B. oleracea* were identified by the same methods shown above, and the corresponding sequences were obtained from the *Brassica* Database (BRAD, http://brassicadb.org/brad, accessed on 10 September 2019). The confirmed NAGs were renamed using the abbreviation of species name, the chromosome position, and the name of *Arabidopsis* homologous genes. e.g., *BnaC03.ASN1*.

### 4.2. Phylogenetic Relationship, Gene Structure and Conserved Motif Analysis

The protein sequences from *A. thaliana* and *B. napus* were subjected to perform multiple sequence alignments using ClustalW tool with default settings [55]. To illustrate the evolutionary relationships of the NAGs, the NJ tree was constructed by MEGA 7.0 software with 1000 bootstraps [56]. The phylogenetic tree was displayed using an online evolview tool (https://evolgenius.info//evolview-v2/#login, accessed on 20 September 2019). The motif-based sequence analysis tool MEME (https://meme-suite.org/meme/, accessed on 20 September 2019) was used to predict the conserved domain of each protein with the following parameters: Classic mode, zero or one; Occurrence per sequence (zoops); Motif count, 10; Motif width, 6 to 50 [57]. Additionally, the exon-intron structure was displayed using the TBtools software [58].

### 4.3. Analysis of Protein Properties and Evolutionary Pressure

The MW, pI, GRAVY value of each protein was predicted using the ProtParam tool (https://web.expasy.org/protparam/, accessed on 28 September 2019) [59]. Subcellular localization was predicted using an advanced protein prediction tool WOLF PSORT (https://www.genscript.com/wolf-psort.html, accessed on 28 September 2019). To analyze gene evolutionary pressure, Ka, KS and Ka/Ks values of homologous genes in *Arabidopsis* and *B. napus* were calculated by TBtools based on aa alignments under the default settings [60].

### 4.4. Chromosome Localization, Gene Duplication and Syntenic Analysis

The NAGs were mapped to the *B. napus* chromosomes based on their physical distances in the general feature format (GFF) genome files, which was downloaded from the *B. napus* genome database [28]. The MapChart v.2.32 software was used to visualize the gene position in chromosomes [61]. Multiple Collinearity Scan toolkit (MCScanX) was used to display collinearity and analyze gene duplication events with default settings [62]. The syntenic map was generated using Circos [63]. To explore the amplification mechanism of NAGs, we performed gene synteny analysis among *Arabidopsis* and *B. napus*, *B. rapa* and *B. oleracea*, and displayed the syntenic relationships with MCScanX [61].

### 4.5. Transcriptional Characterization and Coexpression Network Analysis

To determine the transcriptional characterization of NAGs under diverse nutrient stress conditions, leaf and root samples treated different concentrations of nutrients were collected for transcriptome sequencing. Three biological replicates were analyzed in each treatment. Gene expression values were visualized by TBtools heatmap toolkit. DeGNServer tool (http://plantgrn.noble.org/DeGNServer/Analysis.jsp, accessed on 10 October 2019) was used to identify interaction relationships of gene pairs in the same family based on the transcriptome data [52]. The predicted interaction network was displayed through Cytoscape software [64].

### 4.6. Prediction of Cis-Acting Elements in the Promoter Regions of NAGs

The 2.0-kb upstream promoter sequence of each NAG was extracted by VirtualBox 6.0 with the *B. napus* annotation GFF3 files. Then, the PlantCare database (http://bioinformatics.psb.ugent.be/webtools/plantcare/html/, accessed on 15 October 2019) was employed to predict the putative *cis*-elements [65], and the distribution of the putative cis-elements were visualized by the TBtools heatmap toolkit [58]. The Gene Structure Display Sever 2.0 (http://gsds.cbi.pku.edu.cn/) was used to show phytohormone and abiotic stress response cis-elements [66], and cis-elements were displayed with WordArt (https://wordart.com/, accessed on 15 October 2019).

### 4.7. Prediction of 3D Protein Structure

Protein structure was predicted using the SOPMA online software (https://npsa-prabi.ibcp.fr/cgi-bin/npsa_automat.pl?page=npsa_sopma.html, accessed on 28 October 2019) [67]. We further predicted the tertiary protein structure using the Swiss-Model tool (https://swissmodel.expasy.org/, accessed on 28 October 2019) by homology modeling method [68]. Additionally, the PROCHECK test was used to verify the tertiary protein structure in the PDBsum Generate online software (http://www.ebi.ac.uk/thornton-srv/databases/pdbsum/Generate.html, accessed on 28 October 2019). The tertiary protein structure was displayed by Pymol software [69].

### 4.8. Plant Materials and Treatments

The seedlings of *B. napus* cultivar “ZS11” were germinated in a growth chamber (a 16 h light and 8 h dark photoperiod; 60–80% humidity; 300–320 µmol proton m^−2^ s^−1^ light intensity). Seeds were soaked in deionized water for two days in darkness, and then transferred to a seedling-raising plate filled with 0.5 mM CaCl_2_ solution for three days. The seedlings were then grown in a nutrient solution (pH 5.8) containing 6.0 mM NaNO_3_, 1.0 mM NaH_2_PO_4_·2H_2_O, 2.0 mM MgSO_4_·7H_2_O, 2.0 mM KCl, 3.24 mM CaCl_2_, 46.0 µM H_3_BO_3_, 9.14 µM MnCl_2_·4H_2_O, 0.5 µM Na_2_MoO_4_·2H_2_O, 0.77 µM ZnSO_4_·7H_2_O, 0.32 µM CuSO_4_·5H_2_O and 25.0 µM Fe-EDTA. The nutrient solution was renewed every three days. After nine days of growth, the plants were transferred to nutrient solution free of N, P or K for seven days. The same amount of NaCl were applied instead of NaNO_3_, NaH_2_PO_4_·2H_2_O and KCl in the three nutrient deficiency treatments, respectively [70]. For the ammonium toxicity treatment, plants were treated with nutrient solution containing 6 mM NH_4_Cl instead of NaNO_3_ for 9 days. After treatments, leaf and root were sampled and frozen in liquid N and stored at −80 °C prior to RNA extraction. Three biological replicates were retained for each treatment.

### 4.9. Total RNA Extraction and qRT-PCR Analysis

Total RNA was isolated from the samples using a Eastep^®^ Super total RNA Extraction Kit (Promega Biotech, Beijing, China). Toyobo reverse transcription kit was used to synthesize first-strand complementary DNA (cDNA). Then, cDNA was subjected to qRT-PCR analysis using Hieff^®^ qPCR SYBR^®^ Green Master Mix (Yeasen Biotech, Shanghai, China) on the Quant Studio 6 Flex instrument (Life Technologies, Carlsbad, CA, USA). Each reaction was carried out in triplicate in a reaction volume of 10 µL containing 0.4 µL of gene-specific primers (1.0 µM), 2.0 µL of cDNA, 5 µL of SYBR Green Master Mix, and 2.6 µL of sterile distilled water. The PCR program was as follows: 95 °C for 5 min; 40 cycles of 95 °C for 10 s, 60 °C for 20 s and 72 °C for 20 s; 95 °C for 15 s; 60 °C for 1 min; 95 °C for 10 s. Relative gene expression levels were calculated using the 2^−∆∆Ct^ method [71], with a housekeeping gene *EF1-α* (Accession number: DQ312264) used as an internal control [33]. The gene specific primers used for qRT-PCR were designed on Primer3 (https://bioinfo.ut.ee/primer3-0.4.0/, accessed on 5 November 2019) and listed in Table S6.

### 4.10. Statistical Analysis of Data

Data were analyzed by *t*-test using SPSS 25 software. Significant differences were set to * *p* < 0.05 and ** *p* < 0.01.

## 5. Conclusions

N assimilation efficiency (NAE) contributes largely to plant NUE. The assimilation of N in plants is mediated by many important enzymes including AS, GDH, GOGAT, GS, NiR and NR. The identification of key genes encoding those enzymes is vitally important to manipulate crop NAE. Here, we identified 67 NAGs in *B. napus*, which is an important oil crop grown worldwide. Collinearity analysis indicates that segmental duplication and whole-genome duplication are the primary driving force for the NAG evolution. Functional redundancy and differentiation were predicted based on the gene structure and cis-element analysis. Furthermore, more rapeseed NAGs responded significantly to N stress than K and P limitations. However, some NAGs might be involved in P and K homeostasis in *B. napus*. We further identified 12 hub NAGs in N starvation response based on the expression profiles and coexpression network analysis. In summary, our results laid a theoretical foundation for the follow-up functional study of the key rapeseed NAGs and for developing varieties with enhanced NUE in *B. napus*.

## Figures and Tables

**Table 1:** Copy number variation of the N assimilation related gene family in *Arabidopsis* and three *Brassica* species.

Type	Subfamily	*Arabidopsis thaliana*	*Brassica rapa*	*Brassica oleracea*	*Brassica napus*
AS	*ASN1*	1	2	5	4
	*ASN2*	1	1	0	2
	*ASN3*	1	1	1	2
GDH	*GDH1*	1	1	1	2
	*GDH2*	1	3	4	7
	*GDH3*	1	2	3	5
GOGAT	*GLT1*	1	1	1	3
	*GLU1*	1	1	1	2
	*GLU2*	1	2	2	4
GS	*GLN1*	5	9	9	20
	*GLN2*	1	2	2	4
NiR	*NIR1*	1	2	2	5
NR	*NIA1*	1	3	3	7
	*NIA2*	1	/	/	/
Total		18	30	34	67

## Data Availability

The data presented in this study are available on request from the corresponding authors.

## Supplementary Materials

The following are available online at https://www.mdpi.com/article/10.3390/plants10102160/s1, Figure S1: Chromosomal distribution of the nitrogen assimilation related genes in Brassica napus. (a) Chromosomal distribution in genes among the A subgenome. (b) Chromosomal distribution in genes among the C subgenome. Genes from the same subgroups are indicated by the same color. The MapChart v. 2.32 software is used to draw the map. Figure S2: Identification of the cis-regulatory elements (CREs) in the 2.0-kb promoter regions of 12 core nitrogen assimilation related genes. (a). Over-presentation of the CREs in the promoters of 12 genes. The more the CREs, the bigger the typeface size. (b) Nitrogen, phytohormone, plant development and light responsive cis-elements are showed in the promoter regions of the genes. Different CREs are indicated with different color. Figure S3: Calculated ramachandran plots for modeled 3D structures of 12 core proteins associated with N assimilation. The red, brown and yellow areas represent the preferred, permitted and generally permitted areas. In the graphics, α, β, Lα represent α-helix, β-turn, left-handed α-helix, respectively. Table S1: Characteristics of the NARGs in *Brassica napus* and subcellular localization prediction. Table S2: Motif sequence information of the nitrogen assimilation related genes in *Brassica napus*. Table S3: Non-synonymous (Ka) and synonymous (Ks) nucleotide substitution rates for the nitrogen assimilation related genes in *Arabidopsis* and *Brassica napus*. Table S4: FPKM values of the nitrogen assimilation related genes in rapeseed leaf and root under nitrogen, phosphorus, potassium stresses and ammonium toxicity. Table S5: Predicted secondary structure of 12 core proteins associated with N assimilation in *Brassica napus*. Table S6: Primers used for quantitative real-time PCR in this study.
