# Supplementary material for: Nitrogen Assimilation Related Genes in Brassica napus: Systematic Characterization and Expression Analysis Identified Hub Genes in Multiple Nutrient Stress Responses
Source: Plants (Basel). 2021 Oct 12;10(10):2160. doi: 10.3390/plants10102160 (PMC8539475; doi:10.3390/plants10102160)
Supplement: Supplementary file 1 [file plants-10-02160-s001.zip › Figure S1-S3.pptx]

## Slide 1
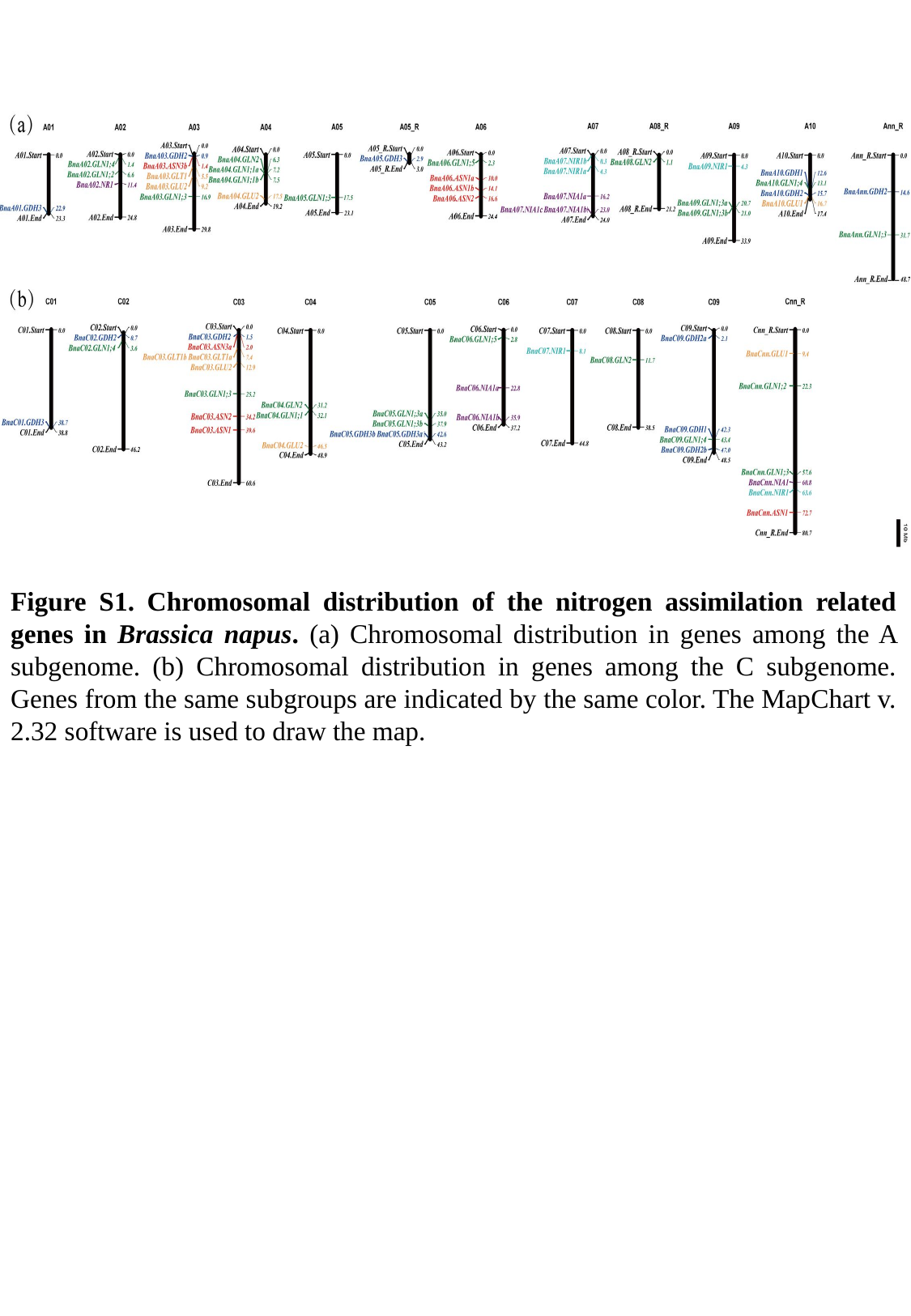

Figure S1. Chromosomal distribution of the nitrogen assimilation related genes in Brassica napus. (a) Chromosomal distribution in genes among the A subgenome. (b) Chromosomal distribution in genes among the C subgenome. Genes from the same subgroups are indicated by the same color. The MapChart v. 2.32 software is used to draw the map.

## Slide 2
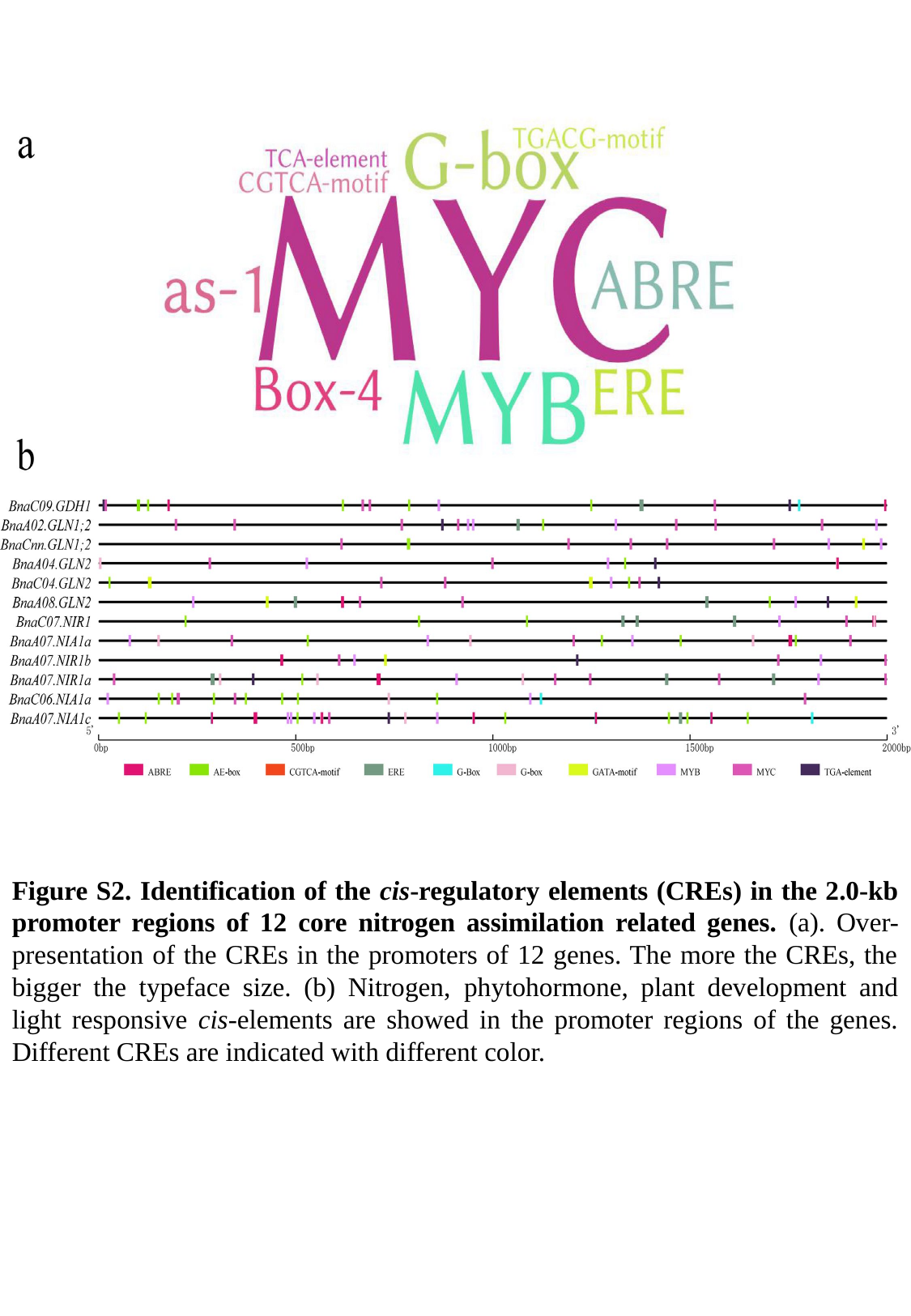

Figure S2. Identification of the cis-regulatory elements (CREs) in the 2.0-kb promoter regions of 12 core nitrogen assimilation related genes. (a). Over-presentation of the CREs in the promoters of 12 genes. The more the CREs, the bigger the typeface size. (b) Nitrogen, phytohormone, plant development and light responsive cis-elements are showed in the promoter regions of the genes. Different CREs are indicated with different color.

## Slide 3
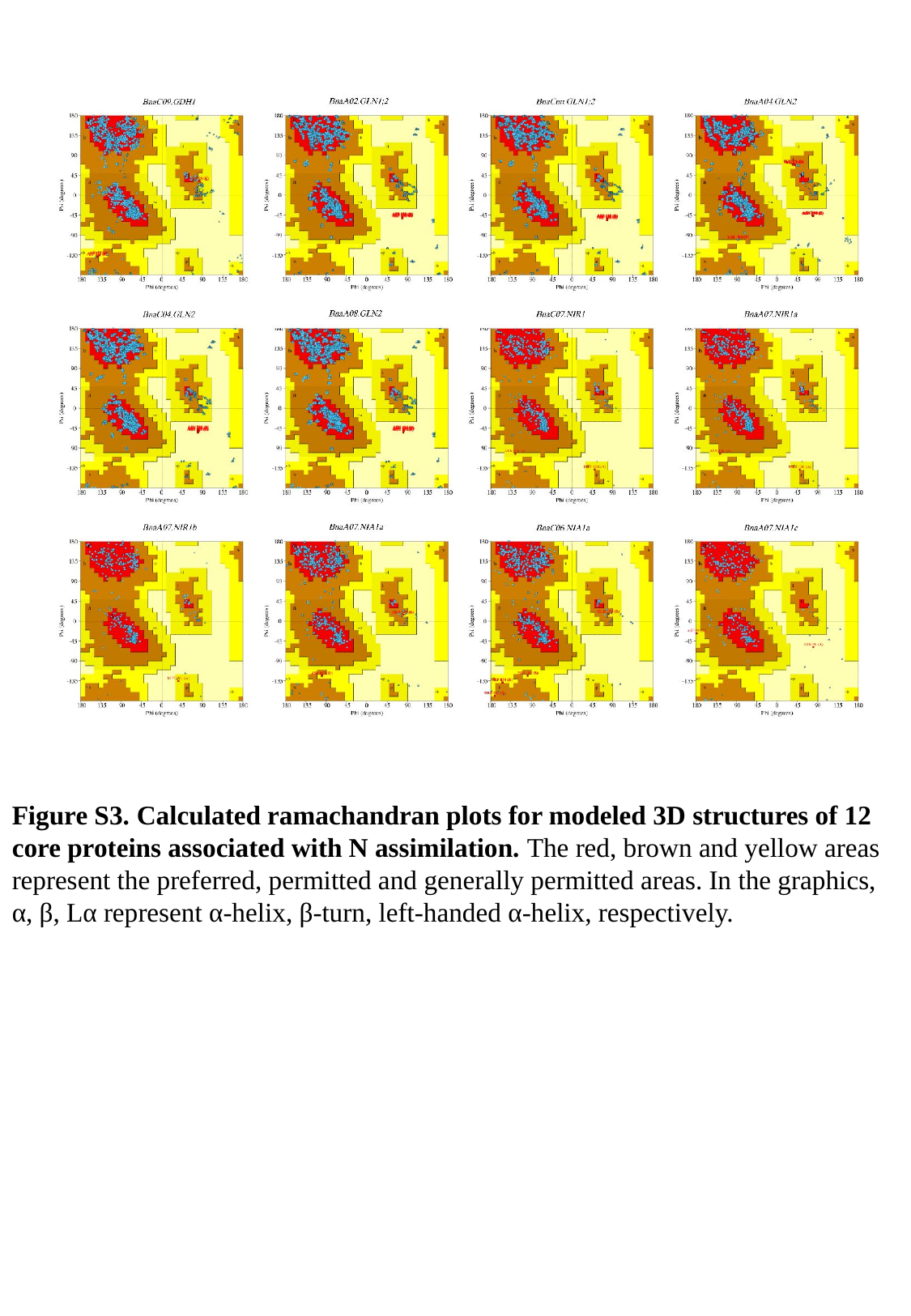

Figure S3. Calculated ramachandran plots for modeled 3D structures of 12 core proteins associated with N assimilation. The red, brown and yellow areas represent the preferred, permitted and generally permitted areas. In the graphics, α, β, Lα represent α-helix, β-turn, left-handed α-helix, respectively.
